# Supplementary material for: D-Pinitol from Ceratonia siliqua Is an Orally Active Natural Inositol That Reduces Pancreas Insulin Secretion and Increases Circulating Ghrelin Levels in Wistar Rats
Source: Nutrients. 2020 Jul 8;12(7):2030. doi: 10.3390/nu12072030 (PMC7400942; doi:10.3390/nu12072030)
Supplement: Supplementary file 1 [file nutrients-12-02030-s001.zip › SUPPLEMENTARY MATERIALS.pdf]

SUPPLEMENTARY

MATERIALS

Supplementary Table 1 (Table 1S). Primer references for TaqMan® Gene Expression Assays (Applied Biosystems).

Gene description	Assay ID	Nº accession GenBank	Amplicon Length
**Target genes**			
Fbp1	Rn00561189_m1	NM_012558.3	77
G6pc	Rn00689876_m1	NM_013098.2	64
Pc	Rn00562534_m1	NM_012744.2	97
Pck1	Rn01529014_m1	NC_005102.4	87
Pklr	Rn01455286_m1	NC_005101.4	58
**Reference genes**			
Gapdh	Rn01775763_g1	NC_005103.4	174
Actb	Rn00667869_m1	NM_031144.2	91

Supplementary Table 2 (Table 2S). Effect of D-Pinitol (100 mg/Kg p.o.) on different metabolites measured in plasma of Wistar male rats at 60, 120 and 240 min post-administration. The values are means ± SEM, (4-5 animals per treated group). Differences between groups were evaluated using one-way Anova + Fisher's LSD test: * P<0.05 vs Control group.

**Plasma metabolic parameters**	**Control**	**60 min**	**120 min**	**240 min**
Uric Acid (mg/dl)	1.8 ± 0.132	1.32 ± 0.139	1.88 ± 0.443	1.24 ± 0.068
Creatinine (mg/dl)	0.525 ± 0.025	0.54 ± 0.024	0.44 ± 0.04	0.54 ± 0.024
Urea (mg/dl)	32.25 ± 2.358	37.4 ± 2.42	41.6 ± 4.155	36.2 ± 3.76
Bilirubin (mg/dl)	0.175 ± 0.025	0.1 ± 0.000 * ↓	0.14 ± 0.024	0.14 ± 0.024
GOT (I.U.)	104.5 ± 12.939	119.0 ± 14.765	127.4 ± 13.923	117.4 ± 12.504
GPT (I.U.)	45.5 ± 8.49	45.8 ± 0.969	59.6 ± 6.608	44.6 ± 4.106

Supplementary Table 3 (Table 3S). Effect of D-Pinitol (500 mg/Kg p.o.) on different metabolites measured in plasma of Wistar male rats at 60, 120 and 240 min post-administration. The values are means ± SEM, (5 animals per treated group). Differences between groups were evaluated using one-way Anova + Fisher's LSD test: * P<0.05, † P<0.01 vs Control group.

**Plasma metabolic parameters**	**Control**	**60 min**	**120 min**	**240 min**
Uric Acid (mg/dl)	1.738 ± 0.107	1.786 ± 0.222	2.025 ± 0.314	1.2 ± 0.195
Creatinine (mg/dl)	0.688 ± 0.061	0.486 ± 0.023 † ↓	0.475 ± 0.065 † ↓	0.45 ± 0.027 † ↓
Urea (mg/dl)	44.143 ± 2.340	34 ± 3.576 † ↓	41.5 ± 1.476	44.25 ± 1.264
Bilirubin (mg/dl)	0.178 ± 0.043	0.1 ± 0.027	0.163 ± 0.038	0.1 ± 0.000
GOT (I.U.)	219.5 ± 23.291	191.625 ± 28.869	133.143 ± 6.859 * ↓	220.125 ± 35.228
GPT (I.U.)	129.875 ± 17.513	103.375 ± 17.644	74.625 ± 7.653	220.125 ± 35.228 † ↑

LEGEND TO SUPPLEMENTARY FIGURES

Supplementary Figure 1 (Figure 1S): qPCR analysis of: **A)** *Fbp1*, **B)** *Pck1* and **C)** *G6pc* gene expression in liver tissue of Wistar male rats measured at different times (10, 20, 30, 60, 120 and 240 min) after D-Pinitol treatment (100 mg/Kg p.o.). The values are means ± SEM, 4-5 animals per group. Differences between groups were evaluated using one-way Anova + Fisher's LSD test.

Supplementary Figure 2 (Figure 2S): Effect of D-Pinitol (500 mg/Kg p.o.) on ghrelin levels (ng/ml) in plasma of Wistar male rats at 60 and 240 min after administration. Values were measured using a commercial ELISA kit. The values are means ± SEM, (5 animals per treated group). Differences between groups were evaluated using one-way Anova + Fisher's LSD test: * $P<0.05$, ‡ $P<0.001$ vs Control group.

Supplementary Figure 3 (Figure 3S): Western blot analysis of the phosphorylation status of the enzymes ERK1 and ERK2 from liver lysates of Wistar rats treated with 500 mg/Kg of D-Pinitol (p.o.) for 60, 120 and 240 min. **A)** Representative western blot analysis for ERK1 (upper panels) and p-ERK1/ ERK1 ratio and ERK1/adaptin ratio (bottom panels) from liver samples of Wistar rats treated with D-Pinitol for times indicated in figure. The blot shows analysis from 4-5 independent samples from each treatment group. The corresponding expression of adaptin is shown as a loading control per lane. **B)** Representative western blot analysis for ERK2 (upper panels) and p-ERK2/ERK2 ratio and ERK2/adaptin ratio (bottom panels) of liver samples from Wistar rats treated with D-Pinitol at times indicated in figure. The blot shows analysis from 4-5 independent samples from each treatment group. The corresponding expression of adaptin is shown as a loading control per lane. All samples shown in the figure were derived at the same time and processed in parallel in the corresponding blot. The adjustment to digital images did not alter the information contained therein. Differences between groups were evaluated using one-way Anova + Fisher's LSD test.

Supplementary Figure 4 (Figure 4S): Western blot analysis of the phosphorylation status of the enzymes GS and mTOR from liver lysates of Wistar rats treated with 500 mg/Kg of D-Pinitol (p.o.) for 60, 120 and 240 min. **A)** Representative western blot analysis for GS (upper panels) and p-GS/GS ratio and GS/adaptin ratio (bottom panels) from liver samples of Wistar rats treated with D-Pinitol for times indicated in figure. The blot shows analysis from three independent samples from each treatment group. The corresponding expression of adaptin is shown as a loading control per lane. **B)** Representative western blot analysis for mTOR (upper panels) and p-mTOR/mTOR ratio and mTOR/adaptin ratio (bottom panels) of liver samples from Wistar rats treated with D-Pinitol at times indicated in figure. The blot shows analysis from three independent samples from each treatment group. The corresponding expression of adaptin is shown as a loading control per lane. All samples shown in the figure were derived at the same time and processed in parallel in the corresponding blot. The adjustment to digital images did not alter the information contained therein. Differences between groups were evaluated using one-way Anova + Fisher's LSD test: * $P<0.05$ vs Control group.

Supplementary Figure 5 (Figure 5S): A) and **B)**: effect of glucose administrated intraperitoneally at a dose of 2 g/Kg (Glucose Group) and effect of D-Pinitol 500 mg/Kg (orally) plus glucose 2 g/Kg (i.p.) (Glucose + D-PIN Group) on **A)** glucose and **B)** insulin levels in plasma at differents times after administration (30, 60 and 120 min). The values are means ± SEM, 5-6 animals per group. Data were analysed using two-way Anova + Fisher's LSD test: * P<0.05 Glucose Group 30 min vs Glucose + D-PIN Group 30 min. Also, data were analysed using one-way Anova + Fisher's LSD test: # P<0.05 vs Control Glucose Group, ## P<0.01 vs Control Glucose Group, ### P<0.001 vs Control Glucose Group, $ P<0.05 vs Control Glucose + D-PIN Group, $$$ P<0.001 vs Control Glucose + D-PIN Group. **C)** Western blot analysis of the phosphorylation status of the enzyme AKT from liver lysates of Wistar rats treated with D-Pinitol 500 mg/Kg (orally) plus glucose 2 g/Kg (i.p.) at 60 min after administration. Left panels: representative western blot analysis for AKT, right panels: p-AKT/AKT ratio and AKT/adaptin ratio. The blot shows analysis from three representative independent samples from each treatment group. The corresponding expression of adaptin is shown as a loading control per lane. All samples shown in the figure were derived at the same time and processed in parallel in the corresponding blot. The adjustment to digital images did not alter the information contained therein. Differences between groups were evaluated using a Student t-test: * P<0.05 vs Control group, ** P<0.01 vs Control group.

SUPPLEMENTARY FIGURES

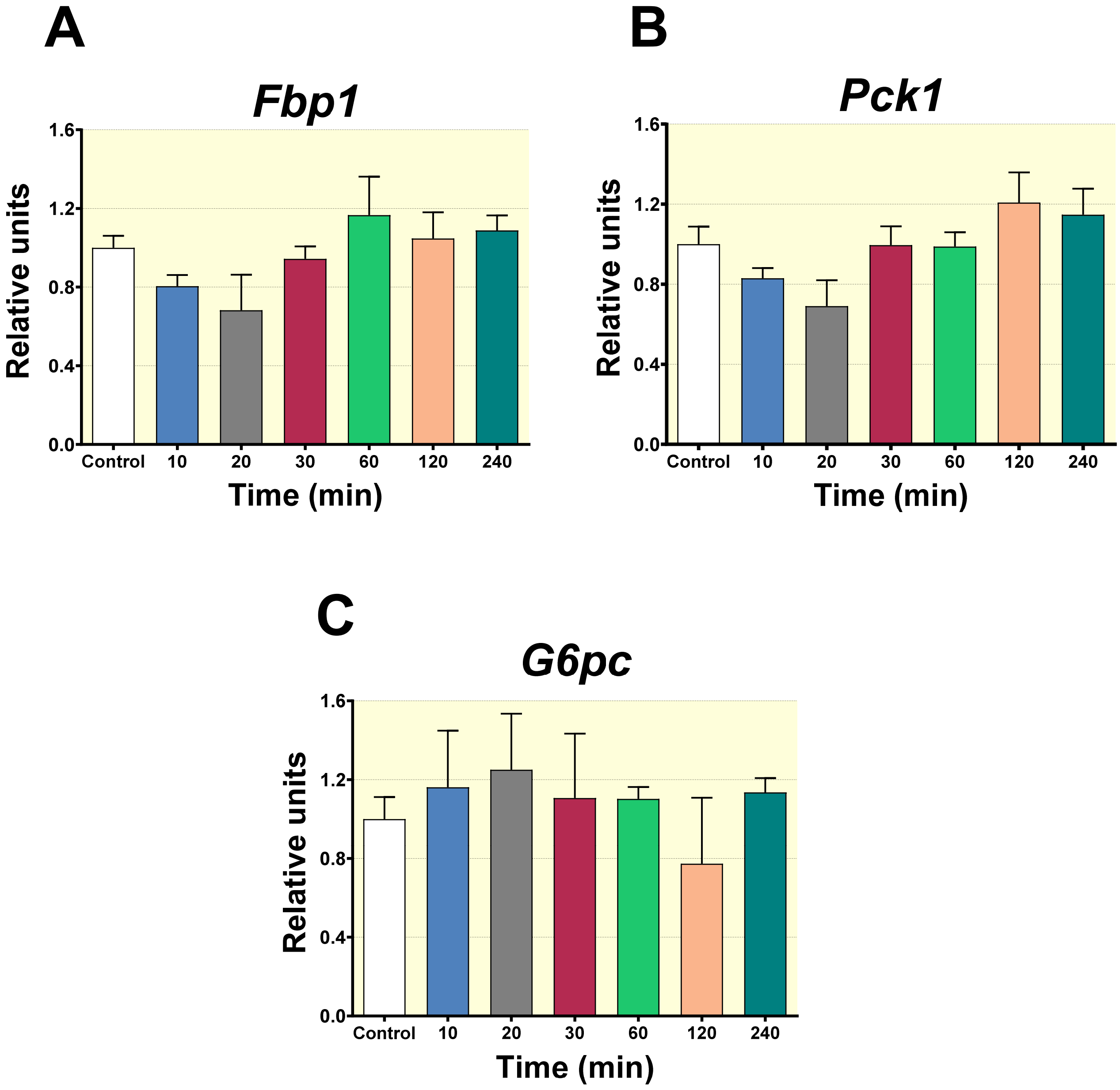
A
Fbp1
Relative units
1.6
1.2
0.8
0.4
0.0
Control
10
20
30
60
120
240
Time (min)
B
Pck1
Relative units
1.6
1.2
0.8
0.4
0.0
Control
10
20
30
60
120
240
Time (min)
C
G6pc
Relative units
1.6
1.2
0.8
0.4
0.0
Control
10
20
30
60
120
240
Time (min)

Figure 1S.

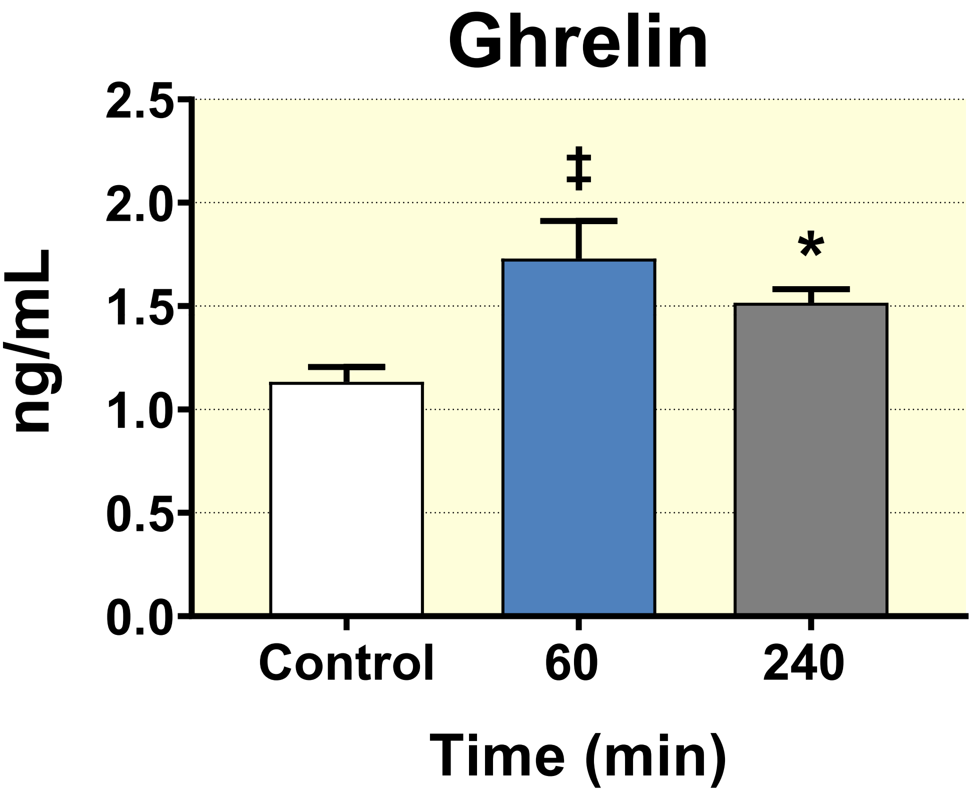

Figure 2S.

A

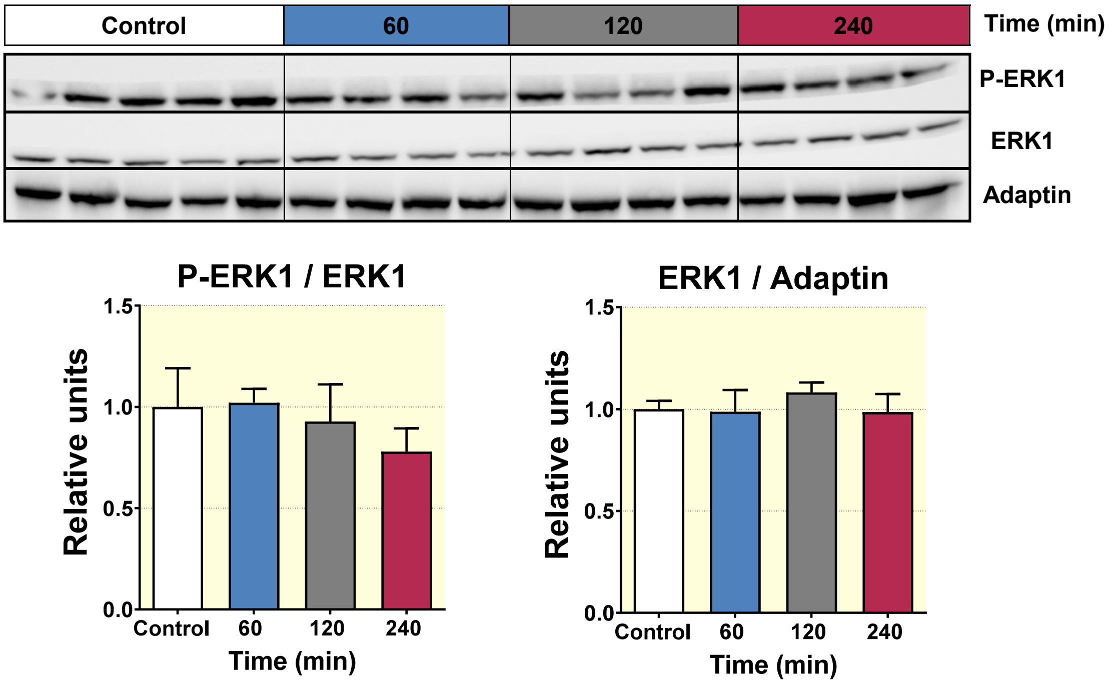

B

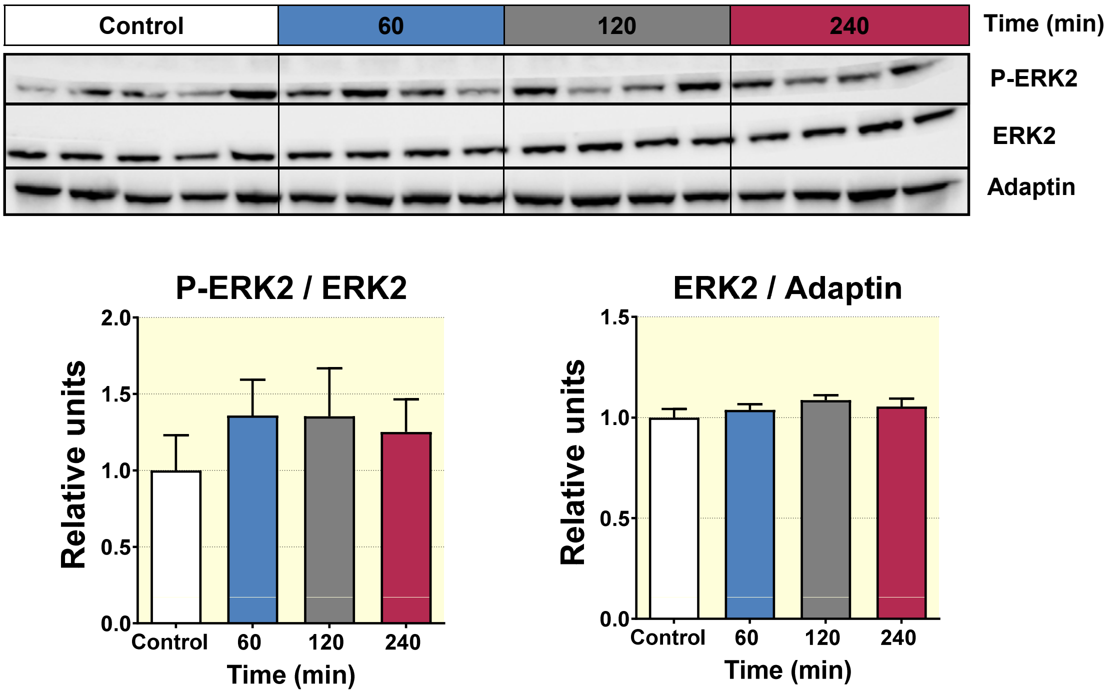

Figure 3S (Pin 500)

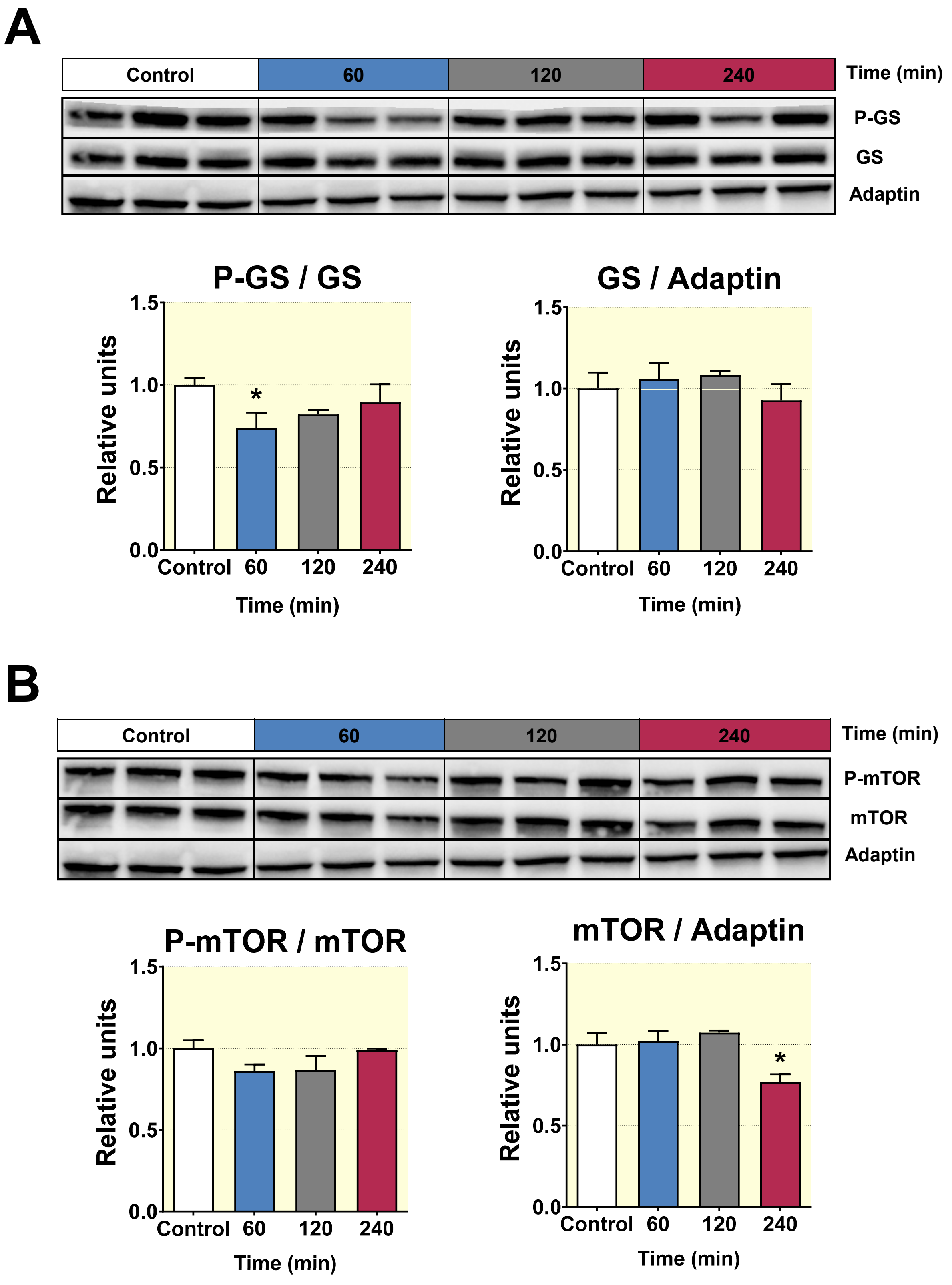
A
Control
60
120
240
Time (min)
P-GS
GS
Adaptin
P-GS / GS
GS / Adaptin
Relative units
1.5
1.0
0.5
0.0
*
Control
60
120
240
Time (min)
B
Control
60
120
240
Time (min)
P-mTOR
mTOR
Adaptin
P-mTOR / mTOR
mTOR / Adaptin
Relative units
1.5
1.0
0.5
0.0
*
Control
60
120
240
Time (min)

Figure 4S

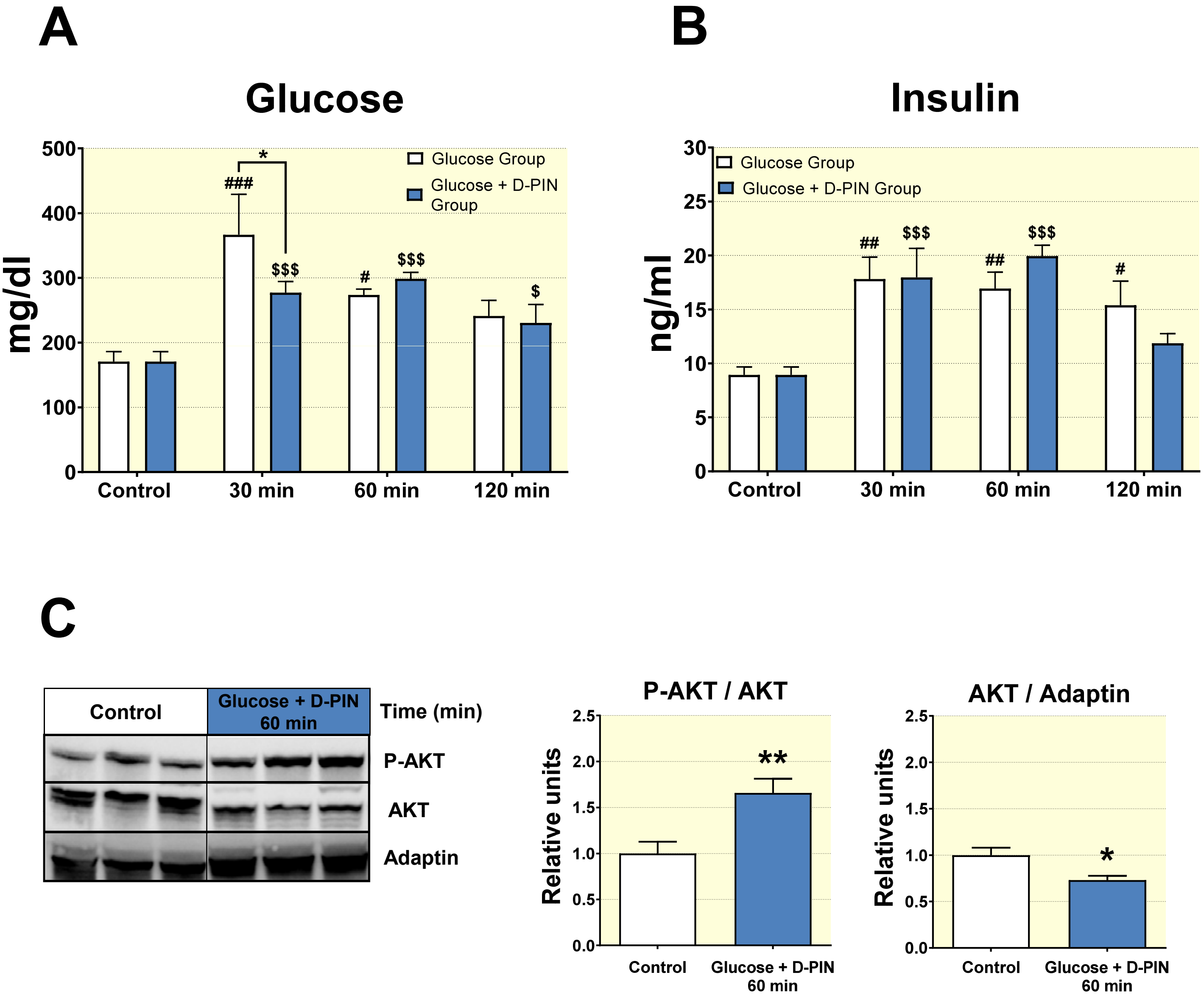

Figure 5S (Pin+Gluc)
